# How the brain constructs dreams

**DOI:** 10.7554/eLife.58874

**Published:** 2020-06-08

**Authors:** Erin J Wamsley

**Affiliations:** Department of Psychology and Program in Neuroscience, Furman UniversityGreenvilleUnited States

**Keywords:** dreaming, sleep, hippocampus, amnesia, episodic memory, memory, Human

## Abstract

Deep inside the temporal lobe of the brain, the hippocampus has a central role in our ability to remember, imagine and dream.

**Related research article** Spanò G, Pizzamiglio G, McCormick C, Clark IA, De Felice S, Miller TD, Edgin JO, Rosenthal CR, Maguire EA. 2020. Dreaming with hippocampal damage. *eLife* **9**:e56211. doi: 10.7554/eLife.56211

Our most vivid dreams are a remarkable replication of reality, combining disparate objects, actions and perceptions into a richly detailed hallucinatory experience. How does our brain accomplish this? It has long been suspected that the hippocampus contributes to dreaming, in part due to its close association with memory: according to one estimate, about half of all dreams contain at least one element originating from a specific experience while the subject was awake (Fosse et al., 2003). Although these dreams are rarely a faithful replication of any one memory, fragments of various recent experiences intermingle with other memories (usually related remote and semantic memories) to create a novel dream. Given all this, one might guess that dreams are created by those regions of the brain responsible for memory. However, studies dating back to the 1960s have suggested that patients with a damaged hippocampus still dream (Torda, 1969a; Torda, 1969b; Solms, 2014) and, somewhat amazingly, such patients can have dreams involving recent experiences of which they have no conscious memory (Stickgold et al., 2000)!

But are the dreams of patients with damage to hippocampus truly ‘normal’? Or alternatively, might such damage, while not preventing dreams, alter the form in which they are expressed? Indeed, there is reason to think that the hippocampus supports crucial aspects of dream construction beyond the simple insertion of memories. Recent work in the cognitive neurosciences has established that the hippocampus, in addition to being involved in the formation of memories, is also part of a brain system that is involved in using memory to construct novel imagined scenarios and simulate possible future events (Hassabis et al., 2007; Hassabis and Maguire, 2009; Schacter and Addis, 2007). As a result, patients without a hippocampus find it difficult to imagine scenes that are coherent, possibly because the hippocampus is responsible for combining different elements of memory into a spatially coherent whole.

Now, in eLife, Eleanor Maguire of University College London (UCL) and colleagues – including Goffredina Spanò as first author – report that the dreams of four amnesia patients lacking a hippocampal memory system do not have the richness of detail found in most dreams (Spanò et al., 2020). Besides reporting substantially fewer dreams than the patients in a control group, the four patients with amnesia also reported dreams that were markedly less detailed: their dreams contained fewer details of spatial location (e.g., descriptions like ‘behind the bar’ or ‘to my left I can see’) and fewer sensory details. These observations support the emerging view that dreams are generated by networks in the brain similar to the networks that are involved in recalling memories and constructing imagined scenarios during wakefulness (Fox et al., 2013; Graveline and Wamsley, 2015). Like memory and imagination, a vivid dream requires the construction of detailed, memory-based imagined scenes – and this process appears to rely on the hippocampus.

These observations partially echo the reports of Clara Torda from more than a half century ago, who characterized the dreams of amnesia patients as ‘shorter’, ‘simpler’, ‘repetitious’ and ‘stereotyped’ (Torda, 1969a). But Torda’s papers were written before the invention of noninvasive methods for imaging the brain, so it is not completely clear which structures may have been damaged in her patients. In contrast, the patients in the work of Spanò et al. all have well-characterized lesion sites with damage restricted solely to the hippocampus. This allows us to confidently attribute their impoverished dreams to the loss of the hippocampus itself, rather than to other regions of nearby temporal lobe which might also have a role in dreaming.

As with many studies of rare neurological patients, the latest work must be interpreted with caution due to the small sample size. For example, patient dreams were not significantly shorter than control dreams, leading to an apparently selective deficit in specific types of details reported (such as spatial details and sensory details), rather than a general deficit in the length of the dream. On average, however, the control dreams contained more than twice the number of informative words as the patient dreams, and the lack of a statistical difference between the two groups may be a mere artifact of the low sample size.

Nonetheless, these observations and a handful of similar studies are helping us to understand how the hippocampus contributes to the dreaming process. The work of Spanò et al. – who are based at UCL, the Royal Free Hospital in London, University Hospital Bonn and the universities of Arizona and Oxford – suggests hippocampal damage disrupts dreaming in ways that mirror how it also disrupts imagination. This suggests that, rather than being an entirely distinct phenomenon, dreaming is a part of a continuum of spontaneous, constructive thought and imagery continuously generated across the sleep and waking states.
